# *Trypanosoma cruzi* Detection in Colombian Patients with a Diagnosis of Esophageal Achalasia

**DOI:** 10.4269/ajtmh.17-0417

**Published:** 2018-02-05

**Authors:** Santiago Panesso-Gómez, Paula Pavia, Iván Enrique Rodríguez-Mantilla, Paola Lasso, Luis A. Orozco, Adriana Cuellar, Concepción J. Puerta, Belén Mendoza de Molano, John M. González

**Affiliations:** 1 Grupo de Ciencias Básicas Médicas, School of Medicine, Universidad de Los Andes, Bogotá, Colombia;; 2 Scientific Research Unit, Hospital Militar Central, Bogotá, Colombia;; 3 Laboratorio de Parasitología Molecular, School of Sciences, Pontificia Universidad Javeriana, Bogotá, Colombia;; 4 Gastrocenter, Investigación y Desarrollo Sistemas Clínicos, Bogotá, Colombia;; 5 Grupo de Inmunobiología y Biología Celular, School of Sciences, Pontificia Universidad Javeriana, Bogotá, Colombia;; 6 Gastroenterology Section, Hospital Universitario Fundación Santa Fe de Bogotá, Bogotá, Colombia

## Abstract

Achalasia is a motility disorder of the esophagus that might be secondary to a chronic *Trypanosoma cruzi* infection. Several studies have investigated esophageal achalasia in patients with Chagas disease (CD) in Latin America, but no related studies have been performed in Colombia. The goals of the present study were to determine the presence of anti-*T. cruzi* antibodies in patients with esophageal achalasia who visited a referral hospital in Bogotá, Colombia, and to detect the presence of the parasite and its discrete typing units (DTUs). This cross-sectional study was conducted in adult patients (18–65 years old) who were previously diagnosed with esophageal achalasia and from whom blood was drawn to assess antibodies against *T. cruzi* using four different serological tests. *Trypanosoma cruzi* DNA was detected by conventional polymerase chain reaction (cPCR) and quantitative polymerase chain reaction (qPCR). In total, 38 patients, with an average age of 46.6 years (standard deviation of ±16.2) and comprising 16 men and 22 women, were enrolled. Five (13.15%) patients were found to be positive for anti-*T. cruzi* antibodies by indirect immunofluorescence assay (IFA), and two patients who were negative according to IFA were reactive by both enzyme-linked immunosorbent assay and immunoblot (5.3%). Parasite DNA was detected in two of these seven patients by cPCR and in one of these by qPCR. The parasite DTU obtained was TcI. In summary, this study identified *T. cruzi* in Colombian patients with esophageal achalasia, indicating that digestive compromise could also be present in patients with chronic CD.

## INTRODUCTION

Chagas disease (CD), which is caused by the intracellular protozoan *Trypanosoma cruzi*, is a major endemic parasitic infection in Latin America. In Colombia, it is estimated that 437,960 individuals are infected and that nearly 11% of the population is at risk of contracting the disease. However, a recent study indicated that only 1.2% of this population has screening coverage.^1^ Although the vector transmission of the parasite is confined to the Latin American continent and certain regions of North America, human migration has spread congenital and transfusion infections to Europe and Asia. Symptomatic chronic infection occurs in approximately 30% of infected patients, in whom the cardiac and digestive systems are the most frequently affected.^2^ The pathogenesis of tissue damage during chronic infection is unclear; however, parasite persistence, cellular tropism, the parasite genotype, and a dysfunctional host immune response have been suggested to participate in the mechanisms that induce damage.^3–6^ During a chronic symptomatic infection, approximately 90% of patients develop cardiomyopathy, 10% develop enteropathy, and a very low percentage of patients exhibit mixed compromise.^2^ The currently identified parasite genotypes, or discrete typing units (DTUs), include seven variants, termed TcI to TcVI and TcBat. Although certain studies have attempted to associate a parasite’s DTU with tissue tropism and clinical presentation, these associations are not completely understood.^5^

Most studies on CD have focused on cardiomyopathy, and several have investigated gastrointestinal complications such as megacolon and esophageal achalasia.^6^ The symptoms of chagasic esophageal achalasia are indistinguishable from those of other causes of esophageal achalasia (i.e., idiopathic); however, a drug provocation test showed greater dysfunction of the esophageal excitatory innervation in patients with CD.^7,8^ In Brazil, the prevalence of esophageal achalasia in *T. cruzi*-endemic areas ranges from 7% to 15.5% in patients who were previously diagnosed with CD, and it is more common in patients less than 40 years of age.^9,10^ According to one study of Latin Americans who migrated to European countries and were diagnosed with *T. cruzi* infection, dysphagia was present in 12% of donors from Spain, and esophageal involvement, assessed by manometry or barium swallowing tests, was present in 11%,^11,12^ whereas in immigrants living in Italy, the prevalence of esophageal achalasia was 1.2%.^13^ In Colombia, only one study has reported mixed cardiac and gastrointestinal compromise due to CD, and genotyping of tissue samples showed DTU TcI in the esophagus and TcII in the heart.^14^

Although chagasic cardiomyopathy has high mortality,^15^ achalasia is highly correlated with a poor quality of life because of impaired esophageal peristalsis, difficulty swallowing, and the need for surgical interventions. Parasite DNA has been detected in tissues from chagasic patients with esophageal compromise.^16–18^ In addition, there is an association between a decrease in the numbers of neurons and glial cells in the myenteric plexus, which innervate the lower esophageal sphincter,^17,19,20^ and the presence of a cellular immune response with a predominance of T cells.^16–21^

The goals of the present study were to determine the presence of anti-*T. cruzi* antibodies in patients diagnosed with esophageal achalasia at a referral hospital in Bogotá, the capital of Colombia, and to detect the presence of circulating parasites in seroreactive individuals by using polymerase chain reaction (PCR).

## MATERIALS AND METHODS

### Study population and ethical considerations.

This descriptive cross-sectional study enrolled patients who attended the Gastroenterology Section at the Hospital Fundación Santa Fe de Bogotá until 2015 and whose information was available from the hospital database beginning in 2006. The study protocol and the informed consent procedure were approved by the Ethical Committees of Universidad de los Andes (190-2012) and Fundación Santa Fe de Bogotá (CCEI 190-2013). The patients, who were between 18 and 65 years of age, signed the informed consent form before participation in this study. These patients, who were diagnosed with or treated for esophageal achalasia, were identified based on electronic medical records (EMRs) and complementary studies (esophageal manometry, chest radiography with barium swallow, and endoscopy). All included patients did not have cardiac compromise. Demographic and epidemiological data, such as age, gender, place of birth and origin, medical history (transfusions or organ transplants), housing conditions, knowledge of the vector, and CD in the family, were obtained.

### Blood sampling and handling.

From each individual, two blood samples were collected by antecubital venous puncture using vacutainer tubes (BD, Franklin Lakes, NJ). One of these samples was collected without anticoagulant to obtain the serum, and the other was collected in a tube containing ethylenediaminetetraacetic acid (EDTA) for DNA extraction. The serum samples were distributed in aliquots and stored at −80°C until used in antibody assays. DNA extraction was performed with a High Pure PCR Template Preparation Kit (Roche^®^, Mannheim, Germany), and the samples were stored at −80°C until PCR assays were performed. A code (Chagas disease: CD-01 to CD-38) was assigned to each patient enrolled in this study to preserve anonymity.

### *Trypanosoma cruzi* antibody detection through an immunofluorescence assay (IFA).

An IFA was conducted using trypomastigotes from a cell culture of the *T. cruzi* TcI DA isolate (MHOM/CO/01/DA), which was obtained from an acutely infected human donor and was maintained as described previously.^22^ Immunofluorescence assay slides containing 5 × 10^4^ methanol-fixed parasites per well were stored under dry conditions at −20°C until use. For the initial screen, the serum was diluted 1:40 (samples and controls) with 1× phosphate-buffered saline (PBS) (0.01 M PBS, pH 7.2). Briefly, the samples were incubated in a humid chamber for 30 minutes in the dark, and after three washes with 1× PBS, anti-human immunoglobulin G (IgG) fluorescein isothiocyanate diluted 1:160 with 1× PBS and the counterstain Evans blue (Sigma-Aldrich, St. Louis, MO) were added. The slides were mounted using low-density Gelvatol (Sigma-Aldrich). Two independent readers evaluated each slide under a fluorescence microscope (Axioskop 40 FL; Carl Zeiss, Oberkochen, Germany) in a blinded manner. Once a positive reaction was detected at 1:40 dilution, the samples were diluted to obtain the anti-*T. cruzi* antibody titer. Positive and negative controls were included, and the assays were performed at least three times.

### *Trypanosoma cruzi* antibody detection using enzyme-linked immunosorbent assay (ELISA).

A commercial kit, Chagas (*T. cruzi*) IgG-ELISA^®^ (NovaTec Immunodiagnostica GmbH; Dietzenbach, Germany), was used to test the samples. The ELISA antigen is a recombinant *T. cruzi* fusion protein (TcF) containing peptides from different *T. cruzi* proteins.^23,24^ The parasite DTUs from which these antigens originated are not described in the insert. The assay was performed according to the manufacturer’s instructions (1:100 serum dilution in duplicate). In addition to the internal controls, external negative and positive controls from Colombian chagasic individuals with cardiomyopathy were included. The absorbance (OD_450_) was measured using a Bio-Rad Microplate Reader 680 (Bio-Rad; Hercules, CA). The control cutoff for the kit was an OD_450_ of 0.306, and the cutoff for negative samples from the laboratory (*N* = 4) was an OD_450_ of 0.253 [mean + two standard deviations (SDs)].

### Chemiluminescent assay (ChLIA) for anti-*T. cruzi* antibodies.

A ChLIA for anti-*T. cruzi* antibodies was conducted using an ABBOTT PRISM Chagas kit (Abbot Laboratories, Abbott Park, IL). This kit uses a recombinant *T. cruzi* protein as an antigen that includes TcF, similar to that used in the ELISA, and three additional peptides from different parasites’ antigens.^25,26^ Diluted samples (1:2 in buffer) were run according to the manufacturer’s instructions. Samples from unexposed individuals (negative control) and from patients with chagasic cardiomyopathy (positive control) were also assessed. A value greater than 1.0 was considered to indicate a positive reaction. The clinical laboratory of the hospital performed this assay in a blinded manner.

### Immunoblot assay for anti-*T. cruzi* antibodies.

An immunoblot was performed using the recombinant TcF antigen from the Chagas IgG LineBlot kit (TRYG2570; NovaTec Immunodiagnostica GmbH). Samples diluted 1:100 were run according to the manufacturer’s instructions. After signal development and drying of the strips, photographs were captured using an iPhone 6 camera (Apple Inc., Cupertino, CA).

### Immunofluorescence assay and ELISA assays with epimastigotes.

All serum samples were tested for IgG anti-*T. cruzi* antibodies at the National Institutes of Health (Instituto Nacional de Salud) in Bogotá, Colombia, a referral center for the diagnosis of CD. The antigen used for both IFA (1% formaldehyde-fixed) and ELISA (total extract) was obtained from epimastigotes of the Colombian DTU TcI strain NV (MHOM/CO/07/NV). The ELISA cutoff for reactivity is considered higher or equal to an optical density of 0.300. For IFA, a result higher or equal to a dilution of 1:32 is considered to indicate reactivity.

### DNA extraction and PCR.

In total, 300 μL of EDTA-anticoagulated blood was used for DNA extraction using a High Pure PCR Template Preparation Kit (Roche). Conventional polymerase chain reaction (cPCR) was run for the β-*globin* gene to assess DNA integrity and to exclude the presence of PCR inhibitors.^27^ Conventional polymerase chain reaction for *T. cruzi* was performed using specific primers targeting a variable region of the mini-circle kDNA, namely, the S35 (AAATAATGTACGGG (T/G) GAGATGCATGA) and S36 (GGGTTCGATTGGGGTTGGTGT) primers, which amplified a 330-bp product. The PCR conditions were described in a previous study.^28^ Quantitative polymerase chain reaction (qPCR) was conducted using the specific primers cruzi 1 (59-ASTCGGCTGATCGTTTTCGA-39) and cruzi 2 (59-AATTCCTCCAAGCAGCGGATA-39) and the probe cruzi 3 (6FAM-59-CACACACTGGACACCAA-39-BBQ), which hybridized to and amplified a 166-bp region in the satellite DNA from the parasite.^29,30^ Each sample was assessed in duplicate using a LightCycler 1.5 Instrument (Roche). The parasitic load was estimated based on a standard curve.^31^ Polymerase chain reaction based on the intergenic region of the mini-exon gene was performed with the primers TCC (5′CCCCCCTCCCAGGCCACACTG3′), TcI (5′GTGTCCGCCACCTCCTTCGGGCC3′), and TcII (5′CCTGCAGGCACACGTGTGTGTG3′) to identify the genotypes in the positive samples; this reaction amplified a 350-bp product for DTU I and a 300-bp product for DTUs II, V, and VI. The PCR conditions were described in a previous study.^32^ Each PCR reaction included a control reaction (water instead of the reaction mixture), a gray control (water instead of DNA in the reaction mixture), a negative control (DNA from a healthy individual), a positive control (*T. cruzi* genomic DNA), and positive controls for TcI and TcII.

### Data analysis and presentation.

Descriptive analyses were used to summarize the patients’ demographic characteristics using percentages, means, and SDs. The data are also reported as proportions. Statistical analyses were not conducted to compare the different groups. All data were stored and analyzed using Microsoft Excel (Microsoft Corp., Redmond, WA).

## RESULTS

In total, 81 patients with a diagnosis of esophageal achalasia were registered in the EMRs through 2015 and 38 agreed to participate in the study. In all, 22 women and 16 men without cardiac compromise and with an average age of 46.6 (SD of ±16.2) years were enrolled. The majority of the patients lived in Bogotá (79%), the capital of Colombia, where *T. cruzi* is not transmitted. Nonetheless, more than half of them were born outside Bogotá (58%), as shown in Table 1. Among the risk factors for CD, 57.9% of the patients had no knowledge of the vector. In addition, most patients had resided in brick houses during their childhood (84.2%), and 100% still resided in this type of housing. Only one individual analyzed had a history of CD in the family (2.6%), and 10 had a history of blood transfusion (26.3%) (Table 2). The most prevalent symptom was difficulty swallowing or dysphagia (97.4%); the frequencies of the symptoms in this cohort of patients are shown in Supplemental Figure 1. The observed signs and symptoms show distributions similar to those described for patients with esophageal achalasia due to other causes.^7^

**Table 1:** Demographic information of the patients who were diagnosed with esophageal achalasia at Fundación Santa Fe de Bogotá University Hospital, Colombia

	Gender		Total
	Male	Female	
Number	16 (42%)	22 (58%)	38
Age, years (mean ± SD)	42.2 (15.6)	52.7 (15.3)	46.6 (16.2)
Place of birth			
Bogotá	4	12	16 (42%)
Outside Bogotá	12	10	22 (58%)
Place of residence			
Bogotá	12	18	30 (79%)
Outside Bogotá	4	4	8 (21%)

SD = standard deviation.

**Table 2:** Risk factors for CD in patients with esophageal achalasia diagnosed at Fundación Santa Fe de Bogotá University Hospital, Colombia

Risk factor	Characteristic	Number	%
Knowledge of or contact with the vector	Yes	15	39.5
	No	22	57.9
	N/A	1.0	2.6
Housing conditions during childhood	Wood	4.0	10.5
	Brick	32	84.2
	Mud	2.0	5.3
Current housing conditions	Wood	0.0	0.0
	Brick	38	100
	Mud	0.0	0.0
History of CD in the family	Yes	1.0	2.6
	No	33	86.8
	N/A	4.0	10.5
History of blood transfusion or organ transplant	Yes	10	26.3
	No	28	73.7
	N/A	0.0	0.0

CD = Chagas disease; N/A = no answer.

### Serological detection of anti-*T. cruzi* antibodies.

The IFA was conducted using a local *T. cruzi* isolate belonging to DTU TcI. Of the samples tested at a 1:40 dilution, five were reactive. Four samples had titers of 1:80 (CD-03, CD-14, CD-18, and CD-32), and one sample had a 1:160 titer (CD-11; Table 3). An ELISA was performed with the recombinant TcF chimeric protein from *T. cruzi*. The reactivity was based on the internal controls, with a cutoff OD_450_ of 0.302. Four Colombian seronegative samples were also included, providing a cutoff OD_450_ of 0.254 (mean + two SDs). Only two individuals analyzed (CD-15 and CD-29) were found to be reactive by ELISA but showed negative results in the IFA. All five individuals who were reactive by the IFA presented negative ELISA results (Table 3). The samples that yielded reactive results by the IFA and ELISA were examined using a ChLIA. This test was chosen because it measures antibodies to the recombinant TcF protein and three additional parasite-derived peptides. Remarkably, no samples were classified reactive through this assay, except for the positive control, which was from a patient with chronic Chagas cardiomyopathy. The same controls (negative and positive) were used throughout the serological tests (ChLIA, ELISA, immunoblot, and IFA) (Table 3). An immunoblot was performed using a commercial kit containing the recombinant TcF antigen with the aim of dissecting the results obtained with the ELISA and ChLIA. A band that was similar to the internal cutoff was observed for two patients (CD-15 and CD-29) who were also reactive by the TcF ELISA. Although the manufacturers regarded bands that exhibited less color than the control as a negative result, a faded band was observed for the recombinant protein from patient CD-14, who presented reactive results by the IFA (Supplemental Figure 2). Finally, both assays (IFA and ELISA) that used epimastigotes to measure IgG yielded negative results for all samples.

**Table 3:** Summary of serological and cPCR assays used to detect anti-*Trypanosoma cruzi* antibodies and *T. cruzi* DNA, respectively, in samples from patients diagnosed with esophageal achalasia

		Assay				
		IFA	ChLIA	ELISA	Immunoblot	cPCR
	Antigen	Trypomastigotes	Recombinant		
	Genotype	(TcI)	Not described			All
	Origin	In-house	Commercial			In-house
Individual analyzed	CD-03	**(+)**	0.11	0.201	(−)	(−)
	CD-11	**(+)**	0.08	0.196	(−)	(−)
	CD-14*	**(+)**	0.07	0.183	Weak	**(+)**†
	CD-15	(−)	0.05	**0.310**	**(+)**	(−)
	CD-18	**(+)**	0.03	0.242	(−)	(−)
	CD-29	(−)	0.04	**0.571**	**(+)**	**(+)**†
	CD-32	**(+)**	0.05	0.130	(−)	(−)
	Negative control	(−)	0.07	0.104	(−)	NA
	Positive control	**(+)**	**12.56**	**2.799**	**(+)**	NA
	Cut-off	1:40 dil	1.00	0.302	Internal control	

CD = Chagas disease; ChLIA: chemiluminescent assay, ABBOTT PRISM Chagas kit, Abbot Laboratories; cPCR = conventional PCR, primers used: S35 (AAATAATGTACGGG (T/G) GAGATGCATGA) and S36 (GGGTTCGATTGGGGTTGGTGT); ELISA = enzyme-linked immunosorbent assay, Chagas (*T. cruzi*) IgG-ELISA NovaTec Immunodiagnostica GmbH; IFA = immunofluorescent assay; IgG = immunoglobulin G; immunoblot, Chagas IgG LineBlot kit (TRYG2570) NovaTec Immunodiagnostica GmbH; NA = not applicable.

Bold values indicate reactivity in the test performed.

* Positive by quantitative polymerase chain reaction.

† Parasite discrete typing unit typed as TcI.

### Detection of parasite DNA by cPCR and qPCR.

Conventional polymerase chain reaction was conducted using primers against the *T. cruzi* kinetoplast DNA in samples from patients who were reactive to any serological test. Parasite DNA was detected in two patients, CD-14 and CD-29; the first individual was classified as reactive by the IFA, and the second individual showed reactive results in the ELISA and the immunoblot against the recombinant TcF protein. Parasite DNA was also detected by qPCR in the CD-14 patient, but the amplification curves showed that the parasitemia load was below the qPCR detection limit (10° parasite equivalents/mL) (Supplemental Figure 3). The parasites from patients CD-14 and CD-29 were typed as DTU TcI.

Of the seven patients (five males) who exhibited reactivity in any serological test or in whom parasite DNA was detected by using PCR, five lived in areas with endemic CD in Colombia, four had knowledge of the vector, two had resided in wooden houses during their childhood, and two had a history of blood transfusion. In summary, two patients who were diagnosed with esophageal achalasia presented reactive serological assay results, and the parasite was detected in their peripheral blood by PCR. In addition, five of the analyzed individuals had inconsistent serological assay results.

## DISCUSSION

Studies of patients with chronic CD have focused on cardiomyopathy, which is the most common complication observed during chronic infection because of its high mortality and socioeconomic impact.^33^ However, several studies from Brazil^9,10^ and from immigrant populations in Spain and Italy^11–13^ have examined the gastrointestinal complications of chronic *T. cruzi* infection. Most of these studies were conducted in patients who had been previously diagnosed with CD and were later examined for symptoms or signs of digestive compromise. The purpose of the present study was to determine the presence of anti-*T. cruzi* antibodies and parasites in individuals with established esophageal disease. Bogotá, the capital of Colombia, is a city at a high altitude (8,660 ft above sea level) where *T. cruzi* is not transmitted, but it is surrounded by municipalities and states located below 6,580 ft where CD is endemic.^34,35^ A high percentage of the population migrates from these places (mainly from rural areas) or travels in search of medical attention.^36,37^ Considering that the majority of individuals analyzed are located in Bogotá, they likely were not continuously exposed to the parasite.^34^ Risk factors for *T. cruzi* transmission were uncommon in our cohort of 38 patients, although they were more frequent in seroreactive patients. Indeed, five of seven seropositive individuals analyzed were born in states with high *T. cruzi* transmission (Boyacá, Cundinamarca, and North Santander), where the primary circulating DTUs are TcI (80.7%) and TcII (7.2%).^35^ Although the prevalence of digestive system compromise in CD varies from 7% to 15%,^9–12^ tests for antibodies against *T. cruzi* are not normally part of the routine exams performed in Colombia on patients with esophageal motility alterations. Interestingly, one study in Mexico showed that among 28 asymptomatic individuals with serological evidence of *T. cruzi* infection, 54% had some form of esophageal motor dysfunction, and one presented with achalasia.^38^ Here, an IFA with trypomastigotes of a Colombian TcI isolate was first used, and reactive samples were further assessed using a commercial ELISA kit (NovaTec Immunodiagnostica GmbH). This ELISA showed good performance in Colombian chronic chagasic patients with OD_450_ values greater than 2.00.^39^ Two achalasia patients for whom negative results were obtained by IFA were reactive by ELISA. The ELISA IgG detection kit (TcF antigen) used in this study has been used to detect antibodies in samples derived from patients in Argentina, Brazil, Chile, and Ecuador and shows good performance and reproducibility.^23,40–44^ Our results are inconsistent with two additional in-house IFA and ELISA assessments from a referral center; despite measuring IgG anti-*T. cruzi*, all samples were negative. In these assays, they used epimastigotes as antigens, and the type of antigen appears to be important in the detection of anti-*T. cruzi* antibodies, particularly in the digestive form of CDs. In one study that used IFA, an antigen from amastigotes detected 100% of Chagas digestive cases compared with 87% of cases with trypomastigotes and only 37% of cases with epimastigotes.^45^ The genetic diversity of *T. cruzi* infection might also influence the sensitivity of the techniques used.^46^ *Trypanosoma cruzi* DTUs are geographically distributed and could be associated with the discordance among the serological tests used to detect *T. cruzi*-specific antibodies.^4,47^ Indeed, at least two reactive tests with different technical principles are needed for diagnosis.^48,49^ Repetitive peptide sequences from *T. cruzi* used in the TcF antigen were originally described in TcII strains [RA strain, clone 2 (PEP-2) and clone 13 (TcD)],^44^ and TcD was also observed in the Tulahuen TcVI strain.^50^ The parasite DTUs from which the TcE and TcLo1.2 sequences were obtained has not been defined.^24^ The third serological assay used (ChLIA) has been tested on samples from patients in Latin American countries, mainly from Argentina, Brazil, Bolivia, and Mexico.^25,26^ Here, none of the samples that yielded reactive results by the IFA and ELISA were reactive in the ChLIA. The additional peptides in the ChLIA, FP3 (TCR27 or FCaBP), FP6 (TCR39 or FRA), and FP10 (SAPA or MAP), were originally identified in a TcI Sylvio X-10/4 clone.^51^ The TcF antigen alone is also included in the immunoblot assay, and two patients with reactive bands were also classified as reactive by the ELISA. All serological assays share TcF as a common antigen; however, the additional peptides in the ChLIA could alter the ability of the antibody to recognize B-cell epitopes. A summary of the data produced with the serological tests used here is shown in Supplemental Table 1.

Regional studies have shown discordance among the assays used to diagnose CD. In Veracruz, Mexico, a study conducted using two in-house ELISAs based on crude epimastigote extracts from the CL-Brener (TcIV) and LJ01 strains (a mixture of TcI and non-TcI isolates) reported a high level of discordance (32%) relative to three commercial kits.^52^ In a cohort of samples from Latin American immigrants to Europe, mainly from Bolivia, 3.3% (165 individuals) yielded discordant results using two ELISAs (one recombinant and one native), and only 28.4% of the discrepancies in the patients’ results were resolved when serological testing was repeated.^53^ In this study, seven patients with esophageal achalasia who had not previously been tested for CD were seroreactive for anti-*T. cruzi* antibodies. In addition, two individuals analyzed with reactive serology exhibited parasitemia. In contrast, a retrospective study in Brazil with 513 patients with esophageal disorders showed 79% seroprevalence for CDs. In addition, among 41 patients with negative or inconclusive serological tests, DNA for *T. cruzi* was amplified in 76% by nested PCR.^54^ As previously shown, the serological assays used for the detection of *T. cruzi* antibodies have high sensitivity and specificity^23–26,40–44^ and showed that individuals with esophageal achalasia and *T. cruzi* serological reactivity had a very low level of antibodies accompanied by low parasitemia, as detected by using PCR. Here, parasite DNA was amplified by cPCR in only two patients, and one had very low but unquantifiable parasitemia. Indeed, the median parasitic load detected by qPCR was 1.33 parasite equivalents/mL in chronic chagasic patients. Unfortunately, this study did not include individuals with digestive compromise.^55^ The low parasitemia and low DNA amplification by PCR could be related to 1) a low tropism of TcI populations by blood, as shown in a mouse model,^56^ 2) the absence of reinfections because the infected individuals live in Bogotá, where there is no transmission of CD,^34^ and 3) the volume of samples used for DNA extraction and the conditions of the PCR. The sensitivity of PCR for *T. cruzi* in blood increases with higher volume collected and with a previous boiling of the lysate when using kDNA-targeted primers because of the linearization of mini-circles.^57,58^

Taken together, these results provide insight into the presence of esophageal compromise in Colombian patients with chronic CD. To overcome difficulties in the diagnosis through serological assay and PCR detection, it is important to consider the use of several tests with similar antigens to the local circulating parasites and to improve the sensitivity of the molecular biology techniques by increasing the volume of blood collected.

## Supplementary Material

Supplemental Figures and Tables.
