# Supplementary material for: Trypanosoma cruzi Detection in Colombian Patients with a Diagnosis of Esophageal Achalasia
Source: Am J Trop Med Hyg. 2018 Feb 5;98(3):717–23. doi: 10.4269/ajtmh.17-0417 (PMC5930867; doi:10.4269/ajtmh.17-0417)
Supplement: Supplementary file 1 [file tpmd170417.SD1.pdf]

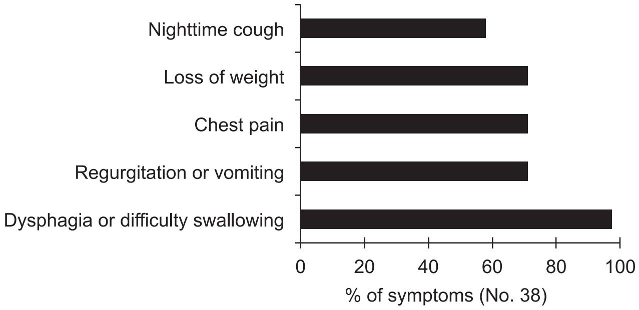

Supplemental Figure 1. Clinical findings for patients with esophageal achalasia. The full bars represent the percentages of each symptom in all participants.

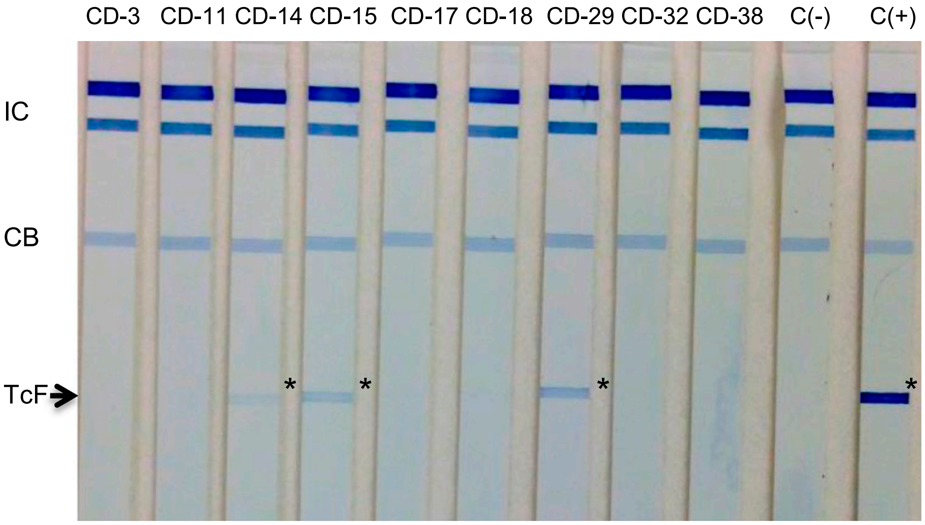

Supplemental Figure 2. Immunoblot assay. The samples assayed are indicated in each lane. An asterisk (*) indicates the presence of any band reactive to the recombinant antigen. Individuals analyzed: CD-03, CD-11, CD-14, CD-18, and CD-32 showed reactive results in the immunofluorescence assay; CD-15 and CD-29 presented reactive enzyme-linked immunosorbent assay results; and CD-17 and CD-38 were not reactive in any serological assay. CB = cut-off band; CD = Chagas disease; IC = internal control; TcF = *Trypanosoma cruzi* fusion protein.

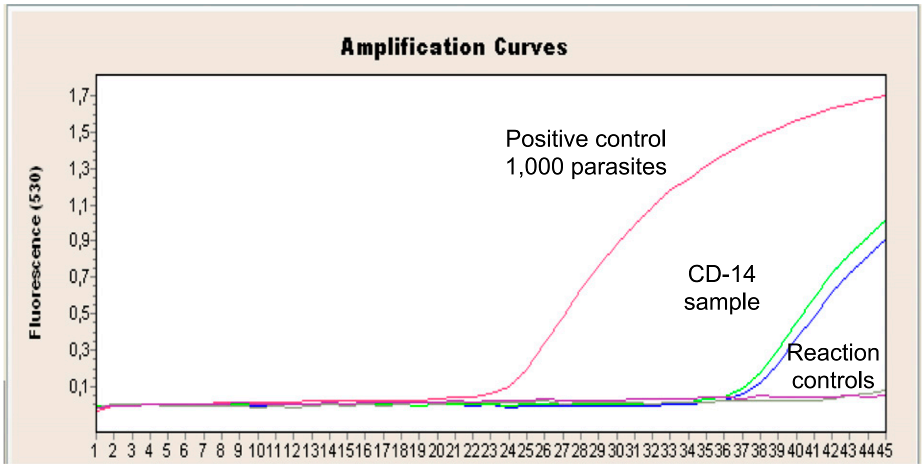

Supplemental Figure 3. Quantitative polymerase chain reaction (qPCR) for *Trypanosoma cruzi* DNA. The qPCR amplification curve is shown. Blue and green lines: duplicate DNA samples from the blood of patient CD-14. Red line: 1,000 parasites/mL. CD = Chagas disease.

Supplemental Table 1

Information from studies done with Chagas serological tests used in this work

Antigen and method	Origin	Samples	Sensitivity %	Specificity %	Note	Source
Clones (number 9) including PEP2 and TcD-immunodot	Argentina, Brazil, and Chile	Chronic infection	ND	ND	Samples tested by IFAT (epimastigotes)	Ibañez et al., 1988 (Ref. 44)
Peptides (number 5) including PEP2 and TcD-IRMA	Chile	Chronic infection	ND	ND	Samples tested by IFAT (epimastigotes) and ELISA (commercial kit)	Vergara et al., 1991 (Ref. 43)
Peptides (number 5) including PEP2 and TcD-ELISA	Chile	Chronic infection	NI	NI	Samples tested by IFAT (epimastigotes)	Vergara et al., 1992 (Ref. 42)
PEP2 and TcD-ELISA	Brazil	Chagasic patients	99.7	99	Samples previously reactive to IFAT, IHA, and ELISA (commercial kit)	Peralta et al., 1994 (Ref. 40)
TcF peptides and TcF-ELISA	Brazil, Ecuador, and USA	Chagasic patients	99.6	99.3	Samples previously reactive to IFAT or ELISA (crude antigen or commercial kit), or RIPA	Houghton et al., 1999 (Ref. 24)
TcF-ELISA	Brazil	Chagasic patients	100	96.6	Treated and untreated patients with parasitemia (xenodiagnosis)	Houghton et al., 2000 (Ref. 41)
TcF-ELISA	Argentina and Brazil	Chagasic patients	100	98.94	Samples previously reactive to IIF, IHA, and ELISA (epimastigotes)	Ferreira et al., 2001 (Ref. 23)
TcF-ELISA*	NI	NI	99	99	Chagas (*Trypanosoma cruzi*) IgG-ELISA*	NovaTec Immunodiagnostica GmbH. Kit's Insert
TcF-ELISA*	Colombia	Chronic infection	96.15	96.55	Samples tested by IFAT and ELISA (epimastigotes)	Llanos et al, 2014 (Ref. 39) Kit Insert
TcF-ELISA*	Mexico	Donors endemic area	ND see agreement test		In house CL-Brener ELISA Kappa Index: 0.205 In house LJ01 ELISA Kappa Index: 0.339	Gúzman-Gómez, et al 2015 (Ref. 52)
TcF + FP3/FP6/FP10 - chemo-luminescence†	Argentina, Bolivia, Brazil, Nicaragua, and Mexico Venezuela	Seroreactive donors	100	99.94	Previously sero-reactive donors by RIPA, IFAT, IHA, or ELISA	Chang et al., 2006 (Ref. 25)
TcF + FP3/FP6/FP10 - Immunoblot†	Argentina, Bolivia, Brazil, Nicaragua, Mexico, Salvador, and Venezuela	Seroreactive donors	100	100	Previously sero-reactive donors by RIPA or ELISA	Cheng et al., 2007 (Ref. 26)

ELISA = enzyme-linked immunosorbent assay; IIF or IFAT = indirect immunofluorescence or immunofluorescence antibody test; IgG = immunoglobulin GIHA = indirect hemagglutination; IRMA = immunoradiometric assay; ND = not done; NI = not indicated; RIPA = radioimmunoprecipitation assay; TcF = *T. cruzi* fusion protein. TcF = TcD + TcE + PEP-2 + TcLo1.2 peptides.

* Chagas (*T. cruzi*) IgG-ELISA, NovaTec Immunodiagnostica GmbH.

† *Trypanosoma cruzi* (*Escherichia coli*, Recombinant) Antigen, ABBOTT PRISM Chagas.
